# Cold-Stimulus Headache in Children and Adolescents

**DOI:** 10.3390/life13040973

**Published:** 2023-04-09

**Authors:** Ilaria Bonemazzi, Maria Federica Pelizza, Giulia Berti, Claudio Ancona, Margherita Nosadini, Stefano Sartori, Irene Toldo

**Affiliations:** Juvenile Headache Center, Department of Woman’s and Child’s Health, University Hospital of Padua, 35128 Padua, Italy

**Keywords:** cold-stimulus headache, ice-cream headache, brain-freeze headache, CSH, children, adolescents

## Abstract

The literature on cold-stimulus headache (CSH) is relatively sparse compared to other primary headache disorders and the studies on the pediatric population are very limited. This systematic review aims to analyze the evidence on CSH in children and adolescents focusing on epidemiology, clinical features, pathogenic mechanisms, and treatments. Our review included 25 studies, among which 9 papers include pediatric cases (4 pediatric samples, 5 mixed samples of children and adults). The aim of this work is to highlight the features of CSH in children and adolescents. In children, the prevalence of CSH is higher than in adults and it is not gender-specific. There is a relevant family history for CSH and the comorbidity with migraine is significant. The triggers and clinical features of CSH due to ingesting a cold stimulus in children overlap with those in adults. CSH due to external application of a cold stimulus (or to environmentally low temperatures) is not studied in children and adolescents. We describe in detail a new pediatric case of CSH triggered by low ambient temperatures; to the best of our knowledge, this represents the first description in the literature. In conclusion, CSH in children is probably underestimated and has peculiar features compared to adults; further studies are needed to better understand its clinical features and pathophysiology.

## 1. Introduction

Cold-stimulus headache (CSH) is a primary headache disorder brought on by a cold stimulus applied externally to the head, ingested, or inhaled [1]. The literature on CSH is relatively sparse compared to other primary headache disorders, as it is a difficult condition to study, mostly because of its short-lasting duration. Moreover, works on the pediatric population are very limited and none of these consider children under 8 years of age.

Nonetheless, it is a common disorder in the general population of all ages, including children [2]. The prevalence varies depending on age, sex, comorbidities, features of the stimulus, and type of exposure. CSH has a higher lifetime prevalence in the pediatric population than in the adult one [3,4,5]. Differently to the adult population in which women have a higher prevalence of CSH than men, there is no significant gender-specific prevalence in children [4,5,6,7].

The prevalence of CSH in children and adolescents, as in the adult population, is higher in patients with a previous history of headaches, in particular migraine (M) [3,4,5,6,7] and headaches attributed to a traumatic injury [3]. It is also higher in patients with a family history of CSH [5].

Concerning triggers, type of exposure, and clinical features, CSH in children and adolescents does not differ from that found in adults.

The pathogenetic mechanism of CSH is still not fully understood and different hypotheses have been put forward, but it is generally believed that local and cerebral vascular changes combined with direct stimulation of cold receptors are involved [2].

The purpose of this systematic review is to analyze the existing evidence regarding CSH in children and adolescents.

## 2. Materials and Methods

In the first part of our work, we searched through PubMed and Mendeley with the following queries: “Cold-stimulus headache”, “Cold temperature headache”, “Ice-cream headache, and “Brain- freeze headache”. The inclusion criteria were the relevance of the paper with the main topic and the presence of a possible explanation of the pathogenesis of a temperature-sensitive headache. We excluded all papers written before 1984. We selected among 36 studies and ended up with 27 studies published between 1984 and 2023: 1 systematic review, 4 standard reviews, 5 letters to the editor with case reports, 5 clinical trials, 6 cross-sectional studies, 3 retrospective studies, 2 observational studies, and 1 case-control study.

We analyzed in detail all the studies focusing on CSH in the pediatric population (9 papers), with the goal of describing the following features: prevalence, predisposing factor, familiarity, clinical features, triggers and type of exposure, possible pathogenetic mechanisms, and treatment. Of the 9 abovementioned studies, 4 consider only pediatric samples, and 5 consider mixed samples of children-adolescents and adults.

To collect the prevalence data, some of these works were based on questionnaires [3,4,5,8,9] or interviews about the headache history of the patients [6,7,10].

We outlined the characteristics of the literature population in Table 1.

In the second part of our work, we reported a clinical case of an 8-year-old patient with CSH triggered by cold environmental temperatures.

## 3. Definition and Diagnosis

CSH is described as a headache brought on by a cold stimulus applied externally to the head, ingested, or inhaled [1]. This definition includes:

- Headache following exposure of the unprotected head to a very low environmental temperature (described also during diving [12], surfing [13], bathing [14] in cold water, skating [15], or receiving cryotherapy [16]);
- Headache occurring immediately after a cold stimulus to the palate and/or posterior pharyngeal wall from ingestion of cold food, drink, or inhalation of cold air, previously known as “ice-cream headache” and “brain-freeze headache”.

These are short-lasting headaches resolved within respectively 30 and 10 min after the removal of the cold stimulus. The diagnostic criteria are reported in Table 2.

## 4. Epidemiology

The lifetime prevalence of CSH varies depending on age, sex, comorbidities, features of the cold stimulus, and type of exposure.

The general lifetime prevalence ranges from 15% to 37% [6,10,17]. In the pediatric population, the lifetime prevalence is higher than in the general population and it is reported in the literature as approximately between 40.6% and 79% [3,4]. Zierz et al. in 2016 [5] compared the lifetime prevalence of CSH in 283 students (10–14-year-old), their parents (401), and 41 teachers with a self-completion questionnaire and found prevalence levels of 62% in the student population and 31% in the adult population. They assume that this might be related to (1) behavioral learning to avoid pain-provoking habits, (2) an age-dependent increased neuronal (e.g., trigeminal) stability against cold stimuli, or (3) the smaller anatomical structure in children, which predisposes them to a quicker local cooling and activation of specific receptors. In the same study [5], they found that, in the student group, there was no age effect on the prevalence of CSH. Instead, Fuh et al. [3] found that the prevalence increased with the grade of school in a study based on patients aged 13 to 15 years. This trend was also found in each gender.

According to most of the studies, there is no significant gender-specific prevalence of CSH in children and adolescents [4,5], while Fuh et al. [3] found that boys have a significantly higher prevalence of ice-cream headaches than girls (43.4% vs. 37.5%). In contrast, women in the adult groups have a significantly higher prevalence of CSH than men [5,6,7].

In most of the studies, children and adolescents with other headaches, as adults, have a significantly higher prevalence of CSH, whereas the absence of any previous headache does not result in an increased prevalence of CSH [3,5,6,7]. Otherwise, Bird et al. [8] found a prevalence of 27% in migraine patients and 40% in the non-cephalalgic population. This fact could be explained by the distribution of the population of that study because the non-cephalalgic population was mostly composed of students, who generally are younger than the general one. 

Comparing patients with a previous history of migraine with those with a previous tension-type headache (TTH), CSH has a higher prevalence in migraineurs (55.2–73.7%) [3,7] than in patients with TTH (23–45.5%) [3,6]. In one single study, it was considered CSH in patients with a previous history of stabbing headache (SH): the prevalence of CSH in this population was about 94% [9].

A previous history of head injury could also be considered as a predisposing factor for CSH when comparing the prevalence of children with (47.3%) to those without (39.2%) [3].

Family history may play a role in CSH. In fact, Ziers et al. [5] found that children had a significant increased risk for CSH when the mother (odds ratio [OR] 10.7) or father (odds ratio [OR] 8.4) had CSH [2]. Other headaches in parents were not significantly associated with CSH in students [5].

The epidemiologic data mentioned above are summarized in Table 3.

## 5. Triggers and Type of Exposure

Several works with a focus on the adult population described CSH following exposure of the unprotected head to very low environmental temperatures (described also during diving [12], surfing [13], bathing [14] in cold water, skating [15], or receiving cryotherapy [16]); there are no reports on children. 

Nevertheless, in children with M, as also described in adults, there is a temperature-sensibility predisposition, which seems to be associated with an earlier age of M onset (20.3 ± 9.8 years vs. 29.1 ± 16.0 years), a longer duration of illness (22.4 ± 13.1 years vs. 14.9 ± 13.2 years), and a higher frequency of M attacks [18]. The study shows that headaches in temperature-sensitive migraineurs are more frequent during the cold periods (or winter), while temperature-non-sensitive patients do not have such association. The decrease in temperature during cold months plays a role in precipitating headache attacks or has a primary effect on headache occurrences [19].

In contrast, another study [20] reported no relationship between exposure to hot or cold weather and the clinical features of headaches among patients with migraine and tension-type headaches.

In children and adolescents, the literature describes few CSH cases only due to ingestion of cold foods or drinks, previously known as “ice-cream headache” and “brain-freeze headache”.

The cold stimulus may be due to the ingestion of cold water [3,5,6], ice cream [4,8,11,21], or ice cubes [3,6,7,9], with different times of placing between the hard palate area and the tongue (30–90 s). 

In adults, CSH triggered by ice water (0 °C) had a shorter latency, different pain character, and higher pain intensity compared to CSH triggered by ice cubes with much lower temperatures (−16° C) [22]. In children, using colder material as a pain stimulus and a prolonged contact time of the cold stimulus increases the rate of CSH [7].

Moreover, the ingestion speed of the cold stimulus seems important in provoking CSH. In fact, Kaczorowski et al. [4] found that in two groups of students who had to eat 100 mL of ice cream at two different speeds (>30 vs. <5 s), the rate of CSH was higher in the accelerated eating group (27%) compared to the cautious one (13%).

## 6. Clinical Features

### 6.1. Type of Pain, Intensity, and Frequency

CSH is characterized by pain described as sharp, intense, and stabbing [1,6,9,21]. However, throbbing pain is also reported in patients with a previous history of migraine [6,7].

The intensity of CSH is referred to mainly as “mild” [3] and “moderate” [6], and is less commonly reported as a headache of “severe intensity” [3,6].

Zierz et al. [5] focused on the frequency of recurrence of CSH with respect to stimulus exposure in a group of students by using a self-administered questionnaire: 21% of cases felt pain every time they were exposed to a cold stimulus, 14% every second to fourth time, and 65% rarely. Differently, Fuh et al. [3] found that 1.4% of their student population always referred CSH after a cold stimulus, 4.3% often, 37.8% sometimes, and 56.6% rarely. Therefore, there are no homogenous findings regarding the frequency of CSH recurrence in the pediatric population.

### 6.2. Localization

According to the IHS classification, the localization of CSH is typically bilateral and mid-frontal, although the pain can be temporal, frontal, or retro-orbital (especially in CSH due to ingestion or inhalation). It can also be unilateral, lateralized to the side of the usual migraine headaches in those who have unilateral headaches [1].

In studies on pediatric populations, the localization of CSH is more common in front of the vertex than behind it. Selekler [9] conducted a cold test for an SH sufferers group. He found that the distribution areas in the head of SH are different from the distribution of those of CSH, and this difference was statistically significant. In particular, 45% of SH cases usually had pain in front of the vertex and 55% behind the vertex; after the cold test, 94% of cases reported pain in front of the vertex and/or on the vertex and 6% behind the vertex.

Specifically, the frontal area of the head [11], the frontotemporal [21], and the temporal one [3,7] are identified as predominant localizations of pain. In a minority of cases, occipital and parietal localizations have been reported [3,6,7]. The pain is mostly bilateral [3,5,6], but in CSH attributed to ingestion or inhalation of a cold stimulus, the pain can be unilateral to the side of the stimulus on the palate [8].

In the study conducted by Selekler et al. in 2004 [7], the pain induced by ice was seen most frequently in the temporal areas in migraineurs (57.1%) than in TTH patients (25%) and it has often been referred to as the part of the head afflicted by the patient’s customary headache. This difference between the groups was statistically significant.

Zierz A.M. et al. [5] found that bilateral CSH was more frequent than unilateral in students without other previous headaches (61 vs. 39%), while for students with other headaches, bilateral and unilateral CSH were distributed equally (49 vs. 51%).

### 6.3. Latency after the Stimulus and Duration of the Headache

The onset of the pain is documented as an early onset [8], typically within 20–30 s after the cold stimulus [3,6,7], but also frequently between 30 and 60–70 s [3,4,5,6,7]. However, De Oliveira et al. [6] found that 41% of the population with CSH started to feel pain between 1 and 8 min after the stimulus.

Concerning the headache duration, the diagnostic criteria define a short-lasting pain, ranging from a few seconds to a few minutes, with resolution within 10 min after the removal of the stimulus for ingestion or inhalation headaches, and within 30 min after the removal of the stimulus for external applications of cold stimulus headaches [1].

Accordingly, we found in all studies a short-lasting duration of the headache [8] ranging from 30 s–1 min to 5 min [3,5,6,21]. The short duration of the headache could be explained by the fact that the available studies on the pediatric populations deal with headaches attributed only to the ingestion of a cold stimulus, and not to an external application.

D. Mitchell [11] reported a case of CSH with other associated symptoms, such as feeling sick and having difficulty seeing, after a consecutive consumption of three large ice creams. In this case, the duration of all symptoms was about an hour, but the duration of the headache was not specified.

We reported the main CSH clinical features in Table 4.

### 6.4. Associated Symptoms

The presence of a cold stimulus applied on the head or over the palate and/or posterior pharyngeal wall could produce other symptoms in addition to headaches.

In the literature, lacrimation, rhinorrhea, conjunctival injection, flashing light dots, and loss of sensitivity in the face have been described in CSH cases [22,23].

For CSH due to ingestion, a history of cold-induced toothache is more common than one of the cold-induced headaches, and palatal or pharyngeal application of ice cream provokes toothache more frequently than headaches [8].

## 7. Pathogenetic Mechanisms

The pathogenetic mechanism of CSH is not completely understood yet. Local and cerebral vascular changes and direct stimulation of cold receptors are the two main hypotheses [2].

In CSH due to ingestion of the cold stimulus, when the palate and/or posterior pharyngeal wall is exposed to a cold substance, this substance may trigger rapid constriction and dilation of vessels with activation of nociceptors of the vessel wall [2].

H. Özyürek et al. measured the middle cerebral arterial (MCA) flow velocities before and while eating ice cream in two children: one with CSH, and one without it. They found a slight decrease in flow velocities in both the child with CSH and the healthy child [21]. In another study conducted on adults, the MCA blood flow velocity was measured while eating ice cream: a decrease in the mean flow velocities was found in subjects who developed a headache, while no changes were observed in those without a headache. Therefore, it is postulated that a decrease in cerebral blood flow is probably secondary to vasoconstriction, which might be important in the development of ice-cream headaches [15,24]. These studies did not explain how cerebral blood flow is mediated intracranially, other than it is hypothesized as a possible mechanism of an overreaction of a vasogenic reflex responding to a small drop in temperature of the carotid blood or a reflex response triggered by the sensation of cold in the palate or oropharynx [24].

According to Hensel et al. [25,26], instead, patients with a headache attack provoked by ice water ingestion had higher MCA mean flow velocity rates than those without headaches. Additionally, patients with a positive ice-cream headache history but negative headache provocation had a moderate mean flow velocity increase. They explain the mechanism of increased cerebral blood flow velocity by a reduction in cerebrovascular resistance secondary to trigeminal-parasympathetic activation. In addition, Mages et al. pointed out that lacrimation occurring during CSH indicates that the trigeminal-autonomic reflex participates in CSH [22].

Burkhart et al. argued that CSH is felt to be a cutaneous sensory response; a reflex response triggered by the sensation of cold in the palate or oropharynx [16].

In line with this, CSH triggered by direct stimulation to the palate (innervated by the trigeminal nerve) may differ from the additional stimulation from the pharynx and esophagus (innervated by the glossopharyngeal and vagus nerve) when swallowing [22]. Therefore, the differences in CSH symptoms could be related to which cranial nerves are activated.

CSH is more common in migraineurs and is often referred to as the part of the head afflicted by the patient’s customary headache. This fact could suggest that there may be segmental disinhibition of central pain pathways in migraineurs, responsible for undue susceptibility to an afferent volley of impulses from the excitation of cold receptors in the oropharynx [9]. Another explanation is that a specific portion of trigeminal pathways may be activated as a reflex for seconds or minutes by a sudden cooling of the pharynx or may discharge migraine headaches for hours. This suggests that hyperexcitability of trigeminal pathways persists between migraine attacks and that periodic discharge of these pathways could initiate migraine headaches [7].

## 8. Treatment

There is no specific treatment for CSH, because of its short-lasting duration. Nevertheless, the triggering factors can be avoided. 

For “ice-cream headache”, some authors suggested that the triggering factors (such as ice cream, ice water, and icy food) could be ingested very slowly, with attempts to minimize rapid exposure of the cold substance to the palate and to the posterior pharynx [4]. Nevertheless, Fuh J. et al. [3] found in their study that the intensity and duration of ice-cream headaches were not different among the students who abstained, decreased, or continued to eat ice cream during a cold test. In addition, they documented that younger students were more likely to decrease or abstain from ice cream because of headaches (from 46.6% at 13 yr. to 33.7% at 15 yr.).

Different studies suggest a different solution depending on the type of stimulus, for example: (a) for CSH due to diving, they suggested minimizing the exposure to cold water and therefore the pain by wearing a neoprene hood [12]; (b) for CSH due to cryotherapy, it is suggested that 1 min of rubbing the face along the distribution of the nerves and vessels before the treatment reduces their hyperexcitability and could eliminate the pain-producing discharges in certain branches of the trigeminal nerve [16].

## 9. Case Report

We describe a case of an 8-year-old patient with CSH triggered by cold environmental temperatures.

He is a healthy patient without a previous history of headaches and without a family history of neurological disorders, in particular headaches.

Since age 5, he has been referred for recurrent attacks of occipital-neck pain of variable intensity (VAS 4- 7/10) occurring with a frequency of 1–2 times/month in summer and 5–6 times/month in autumn and winter, with onset only with exposure to cold temperatures; the pain intensity could often be increased by moderate physical effort. The headache lasted about 5–10 min, with self-resolution. He has no alarm features such as nighttime awakenings or associated symptoms such as sickness, vomiting, phono-, photo-, and osmophobia.

In addition, he denied a stiff neck, dysphagia, hiccups, and deficit of sensitivity and strength. His brain magnetic resonance imaging (age seven) and neurological examination showed no abnormalities.

The referred clinical features of the headache, in particular intensity, short-lasting duration, time of occurrence, and self-resolution fit with all the features of CSH previously described in this review. As an unconventional finding, our patient suffers a headache with localization in a posterior area of the head. Some authors described this localization in a few patients, and some suggested modifying the diagnostic criteria including the affection of the occipital area [27]. Bird et al. tried to explain the occipital pain in CSH saying that the typical anterior pain due to the trigeminal nerve can be followed by an occipitofrontal muscle spasm [8].

## 10. Limitations

There are some potential limitations to this overview. First, a literature search was conducted in the two major electronic databases, PubMed and Mendeley, but no other databases were searched. Therefore, additional studies might have been missed. However, it is noteworthy that they are two of the largest databases of medical studies available. Systematic reviews use a retrospective observational research design, and as such are subject to systematic and random errors. 

A major challenge was to summarize all the features (epidemiology, clinical features, triggers, pathogenic mechanisms, treatment) of CSH despite the clinical and/or statistical heterogeneity of the nine studies that investigated CSH in the pediatric population.

Some of these works collected prevalence data relying on questionnaires; therefore, some values could be altered by the subjectivity of the autofill collection and not by means of a systematic approach.

In addition, the available studies are few and did not find all the same results concerning the same topic (there are different findings, for example, about the frequency of recurrences and the localization of CSH).

## 11. Conclusions

CSH is a complex type of primary headache that is difficult to study, especially in children and adolescents, because of the short duration of the condition and the peculiar features of the pediatric population.

It is an understudied disorder compared to its prevalence; more studies on it in the pediatric age could help to better understand the clinical features and the mechanisms that trigger this condition, particularly concerning CSH induced by a cold stimulus applied externally to the head, as found in our case added to the end of this review.

It would also be interesting to deepen the temperature sensibility in the other cephalalgic patients.

## Figures and Tables

**Table 1:** Demographic and previous history of headaches of the study population (of the 9 papers including pediatric cases).

Study	Number of Patients	Age (Years)	Sex	History of Headache
De Oliveira D. et al., 2012 [6]	414	8–84		^1^ WPH: 35 ^2^ MH: 240
				^3^ TTH: 139
Fuh J. et al., 2003 [3]	8359	13–15	^4^ m 4348.5 +/− 23.5	MPH: ^6^ DNA MH: 526 (m 242, f 284)
			^5^ f 4010.5 +/− 23.5	^7^ NMH: DNA
Kaczorowski M. et al., 2002 [4]	145	10–14		^8^ PH: 37
	
Zierz A. M. et al., 2016 [5]	^9^ A: 442	22–69	m 126	
	^10^ C: 283	10–14	f 157	
Selekler H.M. et al., 2004 [7]	114	14–78	m 26 f 88	MH: 76 (m 9 f 67) TTH: 38 (m 17, f 21)
Bird N. et al., 1992 [8]	120 (70 M + 50 ^11^ s)	15–73		WPH: 19 MH: 72 NMH: 29
Selekler H.M., 2003 [9]	31	16–57		All ^12^ SH MH: 31
Mitchell D., 1984 [11]	1	13		

^1^ WPH = patients without previous headaches. ^2^ MH = migraine headache sufferers. ^3^ TTH = tension-type headache sufferers. ^4^ m = male. ^5^ f = female. ^6^ DNA = data not available. ^7^ NMH = patients with non-migraine headaches. ^8^ PH = periodic headache (not specified). ^9^ A = adults. ^10^ C = children. ^11^ s = students. ^12^ SH = stabbing headache sufferers.

**Table 2:** ICHD-3 diagnostic criteria of CSH [1].

Headache Attributed to External Application of a Cold Stimulus	Headache Attributed to Ingestion or Inhalation of a Cold Stimulus
A. At least two acute headache episodes fulfilling criteria B and C	A. At least two episodes of acute frontal or temporal headache fulfilling criteria B and C
B. Brought on by and occurring only during application	B. Brought on by and occurring immediately after a cold stimulus to the palate and/or posterior pharyngeal wall from ingestion of cold food or drink or inhalation of cold air
C. Resolved within 30 min after removal of the cold stimulus	C. Resolved within 10 min after removal of the cold stimulus
D. Not better accounted for by another ICHD-3 diagnosis.	D. Not better accounted for by another ICHD-3 diagnosis.

**Table 3:** Epidemiologic information of the study population (of the 7 papers including pediatric epidemiology).

Study	Pediatric Prevalence	General Prevalence	Female Prevalence	Male Prevalence	History of Headache Specific Prevalence
De Oliveira D. et al., 2012 [6]		36.7%	39.8%	31.8%	^1^ WPH: 17.1% ^2^ MH: 47.9%
					^3^ TTH: 23% MH + TTH: 38.8%
Fuh J. et al., 2003 [3]	40.6%		37.5%	43.4%	MPH: 29.1% MH: 55.2%
					^4^ NMH: 45.5%
Kaczorowski M. et al., 2002 [4]	79%				
Zierz A. M. et al., 2016 [5]	62%	^5^ A = 31%	^6^ C: 61%	C: 61%	WPH: 55.5% C, 13.1% ^7^ Fa, 24.7% ^8^ Mo
			A: 35%	A: 20%	^9^ PH: 75.5% C, 51.2% Fa, 48.8% Mo
Selekler H.M. et al., 2004 [7]			61.4% MH: 71.6% TTH: 28.6%	53.8% MH: 88.9% TTH: 35.3%	MH + TTH: 59.6% MH: 73.7% TTH: 31.6%
Bird N. et al., 1992 [8]					WPH: 40% MH: 27% TTH: ^10^ DNA
Selekler H.M., 2003 [9]					^11^ SH: 94% MH: DNA

^1^ WPH = patients without previous headaches. ^2^ MH = migraine headache sufferers. ^3^ TTH = tension-type headache sufferers. ^4^ NMH = patients with non-migraine headaches. ^5^ A = adults. ^6^ C = children. ^7^ Fa = fathers. ^8^ Mo = mothers. ^9^ PH = periodic headache (not specified). ^10^ DNA = data not available. ^11^ SH = stabbing headache sufferers.

**Table 4:** Clinical features in the study population (excluding papers with a single-case report).

Study	Frequency	Type of Pain	Intensity	Localization	Laterality	Latency by the Stimulus	Duration
De Oliveira et al. [6]		^1^ Thr: 41.2% ^2^ MH: 43.5% ^3^ TTH: 34.4% ^4^ NThr: 58.8% ^5^ Press: MH: 11.3% TTH: 9.4% ^6^ Burn: MH: 36.5% TTH: 40.6% ^7^ Shock: MH: 8.7% TTH: 15.6%	Mild: 18.3% MH: 14.8% TTH: 21.9%	^8^ FT: 3.7% MH: 14.8% TTH: 9.8%	^12^ U: 22.9% MH: 20.9% TTH: 31.3%	<30 s: 26.8% MH: 27% TTH: 25%	<2 min: 20.3% MH: 20% TTH: 25%
			Moderate: 49% MH: 51.3% TTH: 50%	^9^ F: 47.1% MH: 45.2% TTH: 50%		30–60 s: 32% MH: 32.2% TTH: 37.5%	2–5 min: 68.6% MH: 72.2% TTH: 59.4%
			Severe: 31.4% MH: 32.2% TTH: 28.1%	^10^ T: 34.6% MH: 35.7% TTH: 34.4%	^13^ B: 77.1% MH: 79.1% TTH: 68.8%	1–5 min: 39.2% MH: 39.1% TTH: 34.4%	5–10 min: 6.5% MH: 5.2 TTH: 3.1%
			Unbearable: 1.3% MH: 1.7% TTH: 0%	^11^ O: 4.6% MH: 4.3% TTH: 6.3%		>5 min: 2% MH: 1.7% TTH: 3.1%	>10 min: 4.6% MH: 2.6% TTH: 12.5%
Fuh et al. [3]	Every time: 1.4% Often: 4.3% Sometimes: 37.8% Rarely: 56.6%		Mild: 65.5%	F: 30.1% T: 37.3% ^14^ V: 12.5 % O: 17.5% ^15^ WH: 4.1%	F: U: 9.4%, B: 20.7% T: U: 13.2%, B: 24.1%	20 s	<30 s: 72.3% 30–60 s: 11.7% 1–5 min: 6.8% 5–10 min: 4.5% >10 min: 4.4%
			Moderate: 31.1%				
			Severe: 3.4%				
Zierz et al. [5]	Every time: 21% Every 2–4 times: 14% Rarely: 65%				^16^ WPH: U: 39 %, B: 61%		<10 s: 45% 20–30 s: 77% 30–1 min: 94% >1 min: 6%
					^17^ PH: U: 51%, B: 49%		
Selekler et al. [7]		Thr: MH: 71.4% TTH: 8.3%		F: 17.7% MH: 16% TTH: 14.9% T: 53% MH: 57.1% TTH: 33.3% P: 1.5% MH: 1.7% TTH: 0% V: 11.8% MH: 12.5% TTH: 8.3% O: 5.9% MH: 3.5% TTH: 16.6% ^18^ R: 4.4% MH: 3.5% TTH: 8.3% ^19^ MF: 5.9% MH: 5.3% TTH: 8.3%		0–30 s 31–70 s	
Selekler [9]		^20^ Stab		^21^ fV/V: 94% V/^22^ pV: 3% WH: 3%			

^1^ Thr = throbbing. ^2^ MH = migraine headache sufferers. ^3^ TTH = tension-type headache sufferers. ^4^ NThr = non-throbbing. ^5^ Press = weight/pressure. ^6^ Burn = burning/blasted. ^7^ Shock = shock sensation. ^8^ FT = frontal and temporal region. ^9^ F = frontal and orbital region. ^10^ T = temporal and temple region. ^11^ O = occipital region. ^12^ U = unilateral. ^13^ B = bilateral. ^14^ V = on the vertex. ^15^ WH = on the whole head. ^16^ WPH = patients without previous headaches. ^17^ PH = periodic headache (not specified). ^18^ R = retro-auricular region. ^19^ MF = multifocal. ^20^ Stab = stabbing. ^21^ fV = in front of the vertex. ^22^ pV = posterior area to the vertex.

## Data Availability

The data of the current study (not included in this published article) are available from the corresponding author upon reasonable request.
